# Epidemiology, clinical presentation, treatment, and follow‐up of chronic mercury poisoning in China: a retrospective analysis

**DOI:** 10.1186/s40360-021-00493-y

**Published:** 2021-05-03

**Authors:** Sun Yawei, Long Jianhai, Zhao Junxiu, Peng Xiaobo, Qiu Zewu

**Affiliations:** 1 Department of Chemical Poisoning Treatment, Department of Hematology, Fifth Medical Center of Chinese PLA General Hospital, No. 8 Dong da Street, Fengtai District, 100071 Beijing, China; 2 Pulmonary and Critical Care Medicine, Beijing Tiantan Hospital, Capital Medical University, 100050 Beijing, China

**Keywords:** Toxicology, Nephrology, Neurology, Epidemiology

## Abstract

**Background:**

There are no reports on the incidence of chronic mercury poisoning in a large population in China. This study investigated the epidemiology, clinical manifestations, treatment, and follow-up of Chinese patients with chronic mercury poisoning.

**Methods:**

Data for 288 mercury poisoning patients were collected at our hospital from July 2014 to September 2019, including sex, age, admission time, blood mercury content, urine mercury content, creatinine, urinary mercury/creatinine ratio, 24-h urinary protein levels, electromyography (EMG) findings, renal biopsy, and follow-up. Patient characteristics were evaluated by statistical and correlation analyses.

**Results:**

First, mercury poisoning in China mainly occurred through occupational exposure and the inappropriate use of mercury-containing cosmetics and Chinese folk remedies (CFRs). Second, the most common symptoms were nervous system (50.3 %), kidney (16.4 %) and breathing (8.0 %). Mercury poisoning-induced Nephrotic syndrome (NS) and peripheral neuropathy are common long-term complications. The complications of occupational and cosmetics-induced mercury poisoning are consistent with international belief. However, the NS caused by CFRs is mainly membranous nephropathy and the probability of peripheral neuropathy caused by CFRs is higher than other pathogens. Third, follow-up data shows that 13 patients with EMG-confirmed neurological injury, 10 showed full recovery after 38.50 ± 8.03 months. Furthermore, among 18 patients with NS, 15 had normal urine protein and serum albumin levels after 22.67 ± 10.26 months.

**Conclusions:**

Regulation of skin-lightening cosmetic products, safety surveillance of CFRs, and prevention and control of occupational exposure must be improved to decrease the incidence of mercury poisoning in China.

## Background

Mercury (Hg) poisoning has been a major public health in the world. People showed different clinical manifestations when exposed in different ways to different forms of mercury, such as Hg vapour (Hg^0^) target damage the brain and kidney, inorganic Hg (IHg) target damage the kidney, and organic Hg (OHg, assumed to be methylmercury; MeHg) target damage the brain [1]. The levels of urinary Hg and MeHg in the majority of the general public in China were below the reference value set by the Chinese health authority, except for a few mining areas, due to abundant food resources and low concentration of methylmercury in inland cultured fish. [2, 3]. Epidemiological data show that mercury poisoning mainly results from occupational contact with mercury, abuse of mercury-containing compounds, or the use of skin-lightening cosmetic products [4–6]. Mercury poisoning is caused by inhalating Hg^0^ or eating foods polluted by IHg in a variety of industrial settings, including the chlor-alkali industry ( Hg^0^ ), thermometer ( Hg^0^ ) and fluorescent lamp manufacturing facilities ( Hg^0^ ), as well as chemical processing ( Hg^0^, IHg), and dental practices ( Hg^0^ ) [5, 7]. Mercury poisoning absorbs IHg via the trans-epidermal and trans-appendageal routes, such as mercurous chloride, mercurous oxide, mercuric chloride and ammoniated mercury [8], following the use of skin-lightening creams has been reported in Africa, Europe, the United States, Mexico, Australia, and Hong Kong [8]. Analysis of 19 creams from China, Thailand, and Vietnam showed that they contained mercury at concentrations ranging from 0.01 to 12,590 mg·kg^− 1^ [9]. Mercury-containing compounds have been used for centuries for commercial and medical purposes and are a common constituent of traditional Chinese medicines (TCMs), such as HgS [10]. Unlike TCMs, which are formally approved medical treatments and have reasonably safe levels of bioaccessible Hg [11], Chinese folk remedies (CFRs) induce mercury poisoning via the inhale Hg^0^ or oral HgS routes which involve an informal use of TCMs, rely mostly on experience rather than on formal teachings, and have not been approved by the government [12]. Although incidents of mercury poisoning in China have been occasionally reported domestically and abroad [13, 14], there have been no clinical studies involving a large sample size to date. In the present study, we carried out a retrospective analysis of the clinical manifestations, treatment, and follow-up of Chinese patients with mercury poisoning. Our findings provide important information for clinicians and health authorities on the pathogeny of mercury poisoning in China.

## Methods

### Patients

**Fig. 1:**
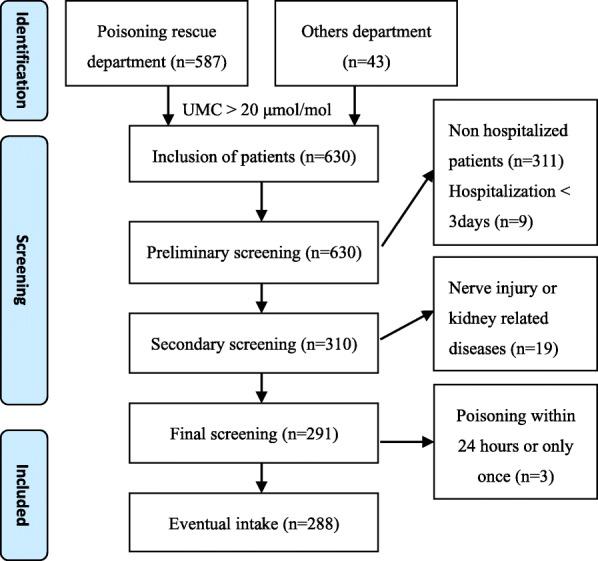
Flow chart of patient screening in this study. UMC, urinary mercury/creatinine

This study was a single-center, retrospective analysis conducted at a 52-bed poisoning treatment center in Fengtai District, Beijing, China. Poisoning patients were mainly admitted from northern, central, and eastern China. The STROBE (strengthening the Reporting of Observational studies in Epidemiology) criteria were followed. In this retrospective study, we analyzed the data for patients treated for mercury poisoning between July 2014 and September 2019 at The Poisoning Treatment Department of The Fifth Medical Center of the PLA General Hospital in China. In total, 288 cases were screened according to the flowchart (Fig. 1). The inclusion criteria were as follows: (1) mercury was detected in the blood and urine, and the urinary mercury/creatinine (UMC) ratio was larger than 20 µmol/mol, which was the chronic Hg exposure limit (35.0 µg/g creatinine ≈ 20 µmol/mol) set by the Chinese Occupational Health Standard (WS/T 265–2006) [2]; and (2) at least one of the following symptoms was present: neurasthenia syndrome (dizziness, fatigue, insomnia, dreaming, forgetfulness, and inattention), tremor, limb or trunk pain, oral gingivitis, proteinuria, etc. The exclusion criteria were as follows: (1) diseases that may cause nephrotic syndrome (NS) or peripheral nerve injury, such as systemic lupus erythematosus, hepatitis B or C, or diabetes, as well as trauma-, drug-, or arsenic-induced peripheral nerve injury; and (2) mercury poisoning within 24 h or only once. All study participants provided informed consent, and this retrospective study design was approved by the appropriate ethics review board of our hospital.

### Data collection

Patient data included baseline characteristics (age, sex, and time of admission); test results, including the creatinine level, blood mercury content, urine mercury content [high urine mercury (HUM): UMC > the biological exposure limit of 20 µmol/mol creatinine], and 24-h urinary protein level (reference value < 150 mg/24 h), collected 1 day after admission; and electromyography (EMG) findings, which were based on the first EMG after admission; and renal biopsy which was performed at an external hospital, and no repeated examination was carried out after admission and during follow-up visits. Mercury concentrations were measured by inductively coupled plasma mass spectrometry using an Agilent 7500ce instrument (Agilent, Santa Clara, CA, USA).

### Statistical analysis

Statistical analyses were performed using the STATA v.13.0 software. Categorical data are reported as numbers and differences were evaluated using the χ^2^ test. Continuous variables were assessed for departure from normality using the Shapiro–Wilk *W*-test with α = 0.10. Normally distributed parametric data are presented as the mean ± standard deviation. Parametric data with skewed distributions are presented as the median ± interquartile range (IQR). Differences between groups were compared using the Student’s *t*-test and Kruskal–Wallis test for normally and non-normally distributed variables, respectively. Correlation analysis between multi-factor and peripheral neuropathy: For the categorical data, we did chi-square test. For the continuous variables, we did logistic analysis. Two-sided *p* values are presented, which were considered statistically significant at *p* < 0.05.

## Results

### Baseline characteristics

Between 2014 and 2019, 288 patients (189 females and 99 males) were hospitalized for mercury poisoning. The mean age was 38.1 ± 12.2 years (range: 3–71 years). In 84 patients (29.27 %), the poisoning was related to cosmetics: the products were not registered in the administration for Industry and Commerce; in some cosmetic products, an excessive mercury content was detected at professional institutions; and some users presented a phenomena that deactivating the same cosmetics products for 6 to 12 months, their UMC was keep in the normal range. In 66 patients (23 %), the poisoning was occupational (forestry work, metallurgy, and machine manufacturing), and Hg0 and Hg^2+^ could clearly be detected at the factory. In 46 patients (16 %), the poisoning was related to CFRs; these patients took CFRs during or before the onset of symptoms, mostly for simple, chronic, and benign illnesses, including the management of psoriasis (*n* = 19, 41.3 %), venous thrombosis (*n* = 1), cervical lymphadenopathy (*n* = 1), acne removal (*n* = 2), insomnia (*n* = 4), migraine (*n* = 3), depression (*n* = 1), glioma (*n* = 1), facial paralysis (*n* = 2), allergic purpura (*n* = 1), urticaria (*n* = 2), rubella (*n* = 1), pruritus of the perineum (*n* = 1), and cervical spondylopathy (*n* = 2), but also as an anodyne (*n* = 1) or for health preservation (*n* = 4). Most CFRs contain cinnabar ( HgS ), calomel ( Hg_2_Cl_2_ ), or other mercury-containing Chinese herbal medicines and even directly use mercury beads ( Hg^0^ ) for heating inhalation. In the remaining 92 patients (32 %), the source of poisoning was unclear; however, accessory examination showed HUM, accompanied by systemic symptoms such as headache, dizziness, tremor, or obvious proteinuria (++).

**Fig. 2:**
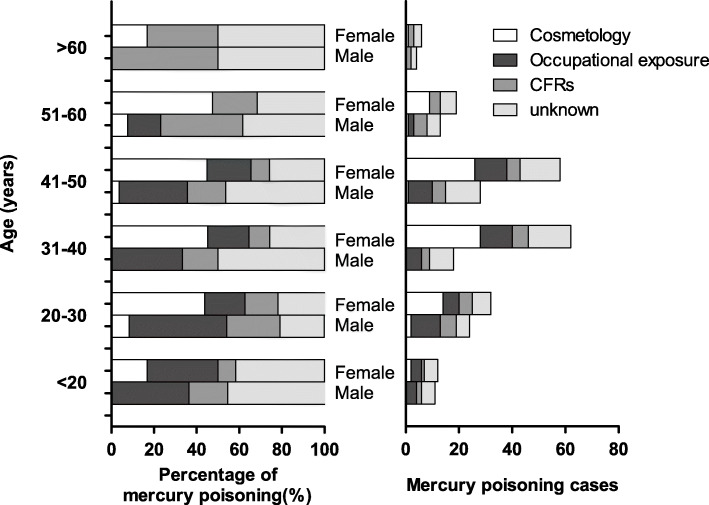
Distribution of mercury poisoning patients by disease etiology, sex, and age. CFRs, Chinese folk remedies. CFRs, Chinese folk remedies

Comparison of patients with occupational mercury poisoning, mercury poisoning caused by cosmetics, and mercury poisoning caused by CFRs showed no obvious differences in their blood mercury content (χ^2^ = 1.05, *p* = 0.590), urinary mercury content (χ^2^ = 3.29, *p* = 0.190), and UMC ratio (χ^2^ = 3.86, *p* = 0.140). The poisoning age distribution showed that most of male patients were aged between 20 and 50 and female patients were aged between 31 and 50. Occupational mercury poisoning is the highest constituent ratio in male (χ^2^ = 3.883, *p* = 0.049), while mercury poisoning was caused by cosmetics in female (χ^2^ = 21.22, *p* < 0.001). Among the patients with mercury poisoning caused by CFRs, there was no sex difference (χ^2^ = 0.004, *p* = 0.949), but there was the first constituent ratio in the male group aged larger than 50 and female group aged larger than 60 (Fig. 2).

### Clinical findings

The median duration of hospitalization was 14 days (range: 3–67 days). There was no correlation between the duration of hospitalization and the source of mercury poisoning (χ^2^ = 1.86, *p* = 0.396). Table 1 shows the symptoms of the 288 patients with mercury poisoning. The most common general symptoms were neurological, nephrological, respiratory, gastrointestinal, and dermatological.

**Table 1:** Symptoms observed in 288 patients with mercury poisoning

Symptoms	Subgroup	Numbers (%)
Neurological		145 (50.3 %)
	Pain in extremities	57 (19.8 %)
	Numbness in extremities	58 (20.1 %)
	Tremor in extremities	10 (3.5 %)
	Dizziness and headaches	23 (8.0 %)
	Myoasthenia	8 (2.8 %)
	Memory problems	3 (1.0 %)
	Insomnia	8 (2.8 %)
Nephrology		76 (26.4 %)
	Edema	45 (15.6 %)
	Foamed urine	17 (5.9 %)
	Nephrotic syndrome	39 (13.5 %)
	Hematuria	2 (0.7 %)
Respiratory		23 (8.0 %)
	Shortness of breath	9 (3.1 %)
	Chest tightness	22 (7.6 %)
Gastrointestinal		15 (5.2 %)
	Pain in throat, chest, or abdomen	9 (3.1 %)
	Nausea and vomiting	6 (2.1 %)
Dermatological		5 (1.7 %)
	Erythematous skin rashes	5 (1.7 %)

### Laboratory findings

**Fig. 3:**
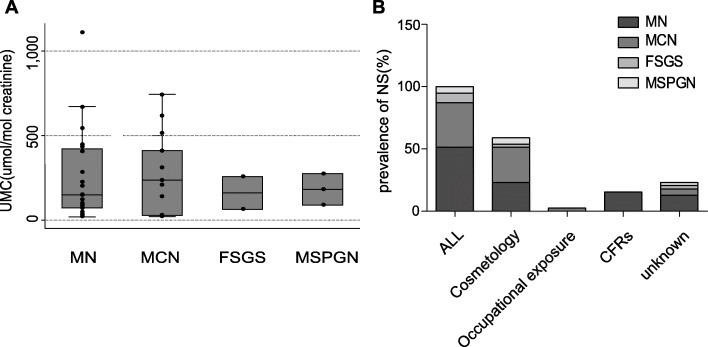
Urinary mercury/creatinine ratio and constituent ratio in 39 cases of mercury poisoning with NS. **a** Distribution of the UMC ratio in accordance with the type of NS. **b** Constituent ratio of different types of NS according to etiology. CFRs, Chinese folk remedies; FSGS, focal segmental glomerulosclerosis; MCN, minimal-change nephropathy; MN, membranous nephropathy; MSPGN, mesangial proliferative glomerulonephritis; NS, nephrotic syndrome; UMC, urinary mercury/creatinine

Table 2 shows the laboratory findings. There were significant differences in the blood mercury concentrations (χ^2^ = 10.77, *p* = 0.005) but no significant differences in the urine mercury content (χ^2^ = 4.68, *p* = 0.100) and UMC ratio (χ^2^ = 3.32, *p* = 0.190) between the patients with NS or peripheral nerve injury and those with non-complication mercury poisoning. Renal biopsy was performed in 39 patients, of which 20 (51.28 %) presented with membranous nephropathy (MN), 14 (35.5 %) with minimal-change nephropathy (MCN), three (7.69 %) with mesangial proliferative glomerulonephritis (MSPGN), and two (5.13 %) with focal segmental glomerulosclerosis (FSGS). The incidences of MN and MCN were higher than those of the other types of NS (χ^2^ = 24.10, *p* < 0.001). There were no differences in UMC ratios among the four types of NS (χ^2^ = 2.12, *p* = 0.548; Fig. 3a). MN was mainly detected in patients for whom the source of mercury poisoning was cosmetic products or improperly used CFRs (χ^2^ = 8.40 > χ^2^ [0.05, 3] = 5.84, *p* < 0.050; Fig. 3b). MCN mainly occurred in patients who used cosmetic products containing mercury (χ^2^ = 22 > χ^2^ [0.05, 3] = 12.92, *p* < 0.050; Fig. 3b). EMG was performed in 39 patients with neuroparesthesia (Table 3). We examined the relationships between the EMG findings and age, sex, duration of hospital stay, pathogeny, blood mercury concentrations, urine mercury concentrations, creatinine, and UMC ratios and found that as the age, the probability of mercury-induced peripheral neuropathy is increased (OR = 1.07, χ^2^ = 3.93, *p* < 0.001). Patients with mercury poisoning caused by improperly used CFRs were more likely to have peripheral neurogenic damage (χ^2^ = 13.60, *p* = 0.003).

**Table 2:** Laboratory examination of 288 patients with mercury poisoning

Laboratory examination	Subgroup	Value		Statistic	P
Blood mercury content ^a^,medain(IQR),µg/L		9.35 (4.20 to 21.00)	I Vs II Vs III	10.77	0.005
	Non-complication, I	8.20 (3.60 to 20.60)	III Vs II	5.26	0.022
	Nephrotic syndrome^d^, II	16.70 (7.80 to 37.80)	II Vs I	10.47	0.001
	Peripheral nerve injury^d^, III	10.20 (5.20 to 17.20)	III Vs I	0.35	0.554
Urine mercury content ^a^,medain(IQR),µg/L		13.50 (5.10 to 29.60)	I Vs II Vs III	4.68	0.096
	Non-complication, I	13.25 (4.9 to 29.1)	III Vs II	4.18	0.041
	Nephrotic syndrome, II	20.9 (8.3 to 37.8)	II Vs I	3.17	0.075
	Peripheral nerve injury, III	9.5 (4.9 to 20)	III Vs I	0.93	0.335
Creatinine,medain(IQR),µmol/L		56 (49 to 66)			
UMC^a^,medain(IQR),µmol/molb		128.57 (40.35 to 277.22)	I Vs II Vs III	3.32	0.19
	Non-complication, I	125.18 (37.68 to 269.84)	III Vs II	2.63	0.105
	Nephrotic syndrome, II	205.38 (70.34 to 411.36)	II Vs I	2.89	0.089
	Peripheral nerve injury, III	221.95 (93.51 to 472.06)	III Vs I	0.11	0.746
24-h urinary protein, median (IQR), g/24 h		0.42 (0.26 to 2.75)			
Renal biopsy^b^, n(%)^c^		39 (13.5 %)		24.10	< 0.001
	MN	20 (51.3 %)			
	MCN	14 (35.9 %)			
	MSPGN	3 (7.7 %)			
	FSGS	2 (5.1 %)			
EMG^b^, n(%)		39 (13.5 %)		6.79	0.036
	Neuropathy	33			
	Myopathy	1			
	FW	5			

*EMG* electromyography, *FSGS* focal segmental glomerulosclerosis, *FW* F-wave, *IQR* interquartile range, *MCN* minimal-change nephropathy, *MN* membranous nephropathy, *MSPGN* mesangial proliferative glomerulonephritis, *UMC* urinary mercury/creatinine

^a^One-way analysis of variance by ranks (Kruskal-Wallis Test)

^b^χ^2^ test

^c^Subgroup's constituent ratio

^d^Nephrotic syndrome's diagnosis was established by renal biopsy and nerve injury's diagnosis was established by EMG

**Table 3:** Correlation analysis between multi-factor and peripheral neuropathy

Peripheral neuropathy	OR	Statistic	p
Age	1.07	3.93	< 0.001
Sex		0.05	0.83
Pathogeny		13.6	0.003
Urine mercury		1.18	0.239
Blood mercury		0.24	0.812
Urine protein		-0.12	0.904
Creatinine		-0.75	0.452
UMC ratio		1.16	0.247
Hospitalization		-0.58	0.56

*EMG* electromyography, *OR* odds ratio, *UMC* urinary mercury/creatinine

### Diagnosis and treatment

All 288 patients were diagnosed with mercury poisoning based on the clinical manifestations and UMC values and underwent symptomatic treatment, which included intramuscular injection (0.25 g twice a day) of sodium 2,3-dimercapto-1-propanesulfonate (DMPS) for 3 consecutive days, followed by 4 days of intermittent treatment, for mercury excretion, and detecting once UMC ratio after twice courses. Combining some symptomatic treatments such as administration of calcium carbonate (3 g twice a day orally), Shenkang injection (100 mL once a day as an intravenous infusion), painkillers (10 mg of tramadol once or twice a day or 5 mg of oxycodone), glucocorticoids (prednisone), and neurotrophic drugs (20 µg/day mouse nerve growth factor for injection, 1.5 mg/day cobamamide, or 0.5 g/day citicoline sodium). For treatment of the 39 patients with NS, prednisone was administered at 1 mg/kg/day for 8 weeks, followed by a progressive decrease by 5 mg (1 tablet) every 2 weeks, based on the 24-h urinary protein level. Prednisone administration usually resulted in a rapid (within 2–3 days) improvement of the patient’s clinical status, with a decrease in fever. In the cases with nerve injury, a neurotrophic factor was administered to reduce the myelin swelling and prevent the nerve fiber degeneration.

### Follow‐up

Some patients were from underdeveloped regions, of which 280/288 patients were rechecked within 6 months to 1 year after discharge, and their UMC values were found to be in the normal range (outpatient query system). Unfortunately, after the first re-examination, most patients did not continue to seek treatment and were followed up via telephone calls in September 2020. Only 249 patients were successfully evaluated in the second follow-up, with the median follow-up time of 46 months (IQR: 30–54 months). Among the patients without complications, 218 reported no abnormal symptoms. For patients with NS diagnosed by biopsy, the last 24-h urine protein report was sent via WeChat or a multimedia message as an image to the mobile phone. Of the 18 patients with NS, who successfully completed follow-up visits, 10 patients were diagnosed with MN, six patients with MCN, and one each patient with FSGS and MSPGN. Two patients with MN and one with MCN did not undergo urinary protein re-examination because of the lack of symptoms. For the other NS patients, their urinary protein and serum albumin levels recovered to normal ranges after 22.67 ± 10.26 months. None of the patients underwent a second renal biopsy. For patients with nerve damage, diagnosed by EMG, their symptoms were carefully examined through phone call inquiries, and their latest rechecked EMG reports were sent via WeChat as an image. Of the 13 patients with EMG-confirmed neurological injury, who were followed up, 10 patients showed full recovery after 38.50 ± 8.03 months (EMGs of eight patients confirmed recovery, and two patients confirmed that they had no discomfort), two showed improvements relative to their previous state, and one showed no change.

## Discussion

We report the epidemiology, treatment, and prognosis of patients admitted with mercury poisoning to a hospital in China. We mainly clarified the following three aspects: (1) different mercury poisoning-induced pathogenies show sex and age difference; (2) there was a certain relationship between different pathogenies and mercury poisoning-induced complications; and (3) a clear diagnosis and timely treatment of mercury poisoning are necessary.

The gender and age distribution of occupational mercury poisoning patients are as described above, but most of the occupational exposure are hospitalized in occupational hospitals throughout the country, so the distribution is worth further discussion. Meanwhile, we report that mercury-containing cosmetic products are the main cause of mercury poisoning in females. Mercury is absorbed by the skin [8] and can cause many complications such as nephrotic syndrome and peripheral nerve injury. Unfortunately, skin-lightening creams containing inorganic mercury are widely used by dark-skinned people to achieve a lighter skin tone [9, 15]. In China, TCM can be confused with CFRs. Most patients from this study improperly used CFRs for simple, chronic, and benign illnesses [12], the situation of this study are consistent with above. Meanwhile, this study indicates that the CFRs-induced mercury poisoning is the highest constituent ratio in elderly patients (> 50 years old in male and > 60 years old in female). Before the 1970 s, people had a low level of education and maintained a high trust to CFRs [16].

Long-term mercury exposure causes damage to several organ systems, including the nervous, urinary, digestive, and respiratory systems [17]. Neurological manifestations of inorganic mercury intoxication include weakness, numbness, paresthesia, muscle cramps or atrophy, diminished muscle stretch reflexes, paresis, fasciculations, and sensory loss [18]. The conventional belief is that occupation-induced mercury poisoning is the most common [19–21]. There are only a few domestic and overseas reports related to mercury poisoning caused by TCMs [12]. In this study, EMG showed that most nerve damage in patients was caused by CFRs, likely because occupational disease patients tend to be admitted to occupational disease hospitals. Mercury has a strong affinity for renal tissue which can lead to NS [15, 22–24]. Mercury-induced MN results from long-term use of mercury-containing skin-lightening cosmetics or from occupational contact with mercury [25–27]. Minimal-change glomerular lesions were detected in women in Kenya, who used mercury-containing skin-lightening creams [28], and four cases of MCN were described following the use of mercury-containing skin-lightening cream for 2–6 months [29]. Our study further supported the belief that MN and MCN occur following the use of mercury-containing skin-lightening creams. However, MN was also observed following the inappropriate use of CFRs [11]. The results of this study are consistent with those of most international mercury poisoning cases and reports [30] that used small samples. In addition, it contributes to the treatment and prognosis of mercury poisoning induced by mercury-containing CFRs.

As previously mentioned [31, 32], patients with mercury poisoning are often misdiagnosed in China as having digestive system diseases (acute abdomen and acute gastroenteritis), nervous system diseases (neurasthenia and vegetative nerve functional disturbance), and diseases of the urinary system (acute nephritis and nephritic syndrome). The reasons for the top two misdiagnosed diseases are incorrect history taking by doctors and a shortage of relevant knowledge about mercury poisoning. In China, mercury poisoning can only be definitively diagnosed and treatments provided at prevention and treatment centers for occupational diseases, such as the Centers for Disease Control [12, 32]. Prior to the establishment of poison treatment centers, mercury poisoning cases were mainly treated at the neurology, nephrology, and gastroenterology departments of hospitals, with most patients receiving symptomatic and not etiological treatment [33]. Moreover, although overseas researchers report some long-term follow-up prognoses for nephritic syndrome and peripheral nerve injury caused by mercury poisoning, domestic researchers do not report relevant aspects. Here, the follow-up results showed that the average recovery periods of nerve injury and nephritic syndrome caused by mercury poisoning were 38.5 and 22.67 months after dispelling mercury, respectively. Although there are specific therapeutic drugs and a sound prognosis for mercury poisoning, the state of the illness is prolonged for most patients because of the lack of education in mercury poisoning and a shortage of national poison testing centers, most of which are only used for occupational disease groups. Public awareness of the main causes of mercury poisoning, including the use of cosmetic products and CFRs and occupational exposure, must be increased to relieve the heavy burden of mercury poisoning on both rural Asian communities and their healthcare systems.

This study has several limitations. First, there is a lack of multicenter studies, and there are only a few poisoning treatment centers in China. Although our center is the largest poisoning treatment center, it mainly receives patients from North, Central, and East China, while receiving only few patients from South and West China. Second, owing to the sparse distribution of domestic mercury poisoning centers, low incomes, and high mobility of patients, the second follow-up was only conducted over the telephone, with some proportion lost to follow-up. Third, to comply with China’s national requirements, we adopted the GBZ-2007 National Mercury Poisoning Diagnostic Standards, which have not been updated for a long time and differed from international standards. Fourth, patients with NS caused by mercury poisoning did not receive renal biopsy and could only be evaluated in terms of clinical cure.

## Conclusions

In this article, we report the epidemiology, treatment, and prognosis of patients with mercury poisoning at a hospital in China. Our study found that (1) poisoning in China mainly occurred through occupational exposure and the use of cosmetics and CFRs, as previously reported; (2) mercury poisoning due to occupational exposure was more common in males in the 20–50 year-old age group, while female patients aged 20–50 years were more likely to experience cosmetics-related poisoning. Mercury poisoning due to inappropriate use of CFRs was observed at all ages, regardless of the sex; (3) patients with mercury poisoning caused by the inappropriate use of CFRs were more likely to have peripheral neurogenic damage; NS caused by mercury poisoning was mostly of the MN and MCN types following the use of mercury-containing skin-lightening creams and of the MN type following the inappropriate use of CFRs; (4) most severe complications of mercury poisoning (NS and peripheral nerve injury) can be alleviated by sodium DMPS treatment.

## Data Availability

The datasets during and/or analysed during the current study available from the corresponding author on reasonable request.

## Abbreviations

CFRs: Chinese folk remedies
DMPS: Sodium 2,3-dimercapto-1-propanesulfonate
EMG: Electromyography
FSGS: Focal segmental glomerulosclerosis
HUM: High urine mercury
IQR: Interquartile range
MCN: Minimal-change nephropathy
MN: Membranous nephropathy
MSPGN: Mesangial proliferative glomerulonephritis
NS: Nephrotic syndrome
STROBE: Strengthening the Reporting of Observational studies in Epidemiology
TCMs: Traditional Chinese medicines
UMC: Urinary mercury/creatinine
